# BBD optimized antioxidants of *Crotalaria candicans* and its nanoconjugates, exert potent in vivo anti-biofilm effects against MRSA

**DOI:** 10.1038/s41598-023-43574-0

**Published:** 2023-09-29

**Authors:** Ramya M. Subramani, Robert Lotha, Bhanuvalli R. Shamprasad, Sriram Sridharan, Ravichandran Natesan, Saisubramanian Nagarajan, Arvind Sivasubramanian

**Affiliations:** 1 Department of Chemistry, School of Chemical and Biotechnology, SASTRA Deemed to be University, Thanjavur, Tamil Nadu India; 2 Centre for Advanced Research in Indian System of Medicine, School of Chemical and Biotechnology, SASTRA Deemed to be University, Thanjavur, Tamil Nadu India; 3 Centre for Research on Infectious Diseases, School of Chemical and Biotechnology, SASTRA Deemed to be University, Thanjavur, Tamil Nadu India

**Keywords:** Biofilms, Nanobiotechnology

## Abstract

*Crotalaria* genus is extensively dispersed in tropical and subtropical provinces, and it is found to harbor antioxidant flavonoids. Response surface methodology-based optimization was carried out for the purpose of efficient extraction involving a suitable solvent which can maximize the yield along with higher total phenolic content and total flavonoid content (TFC). Optimization conditions for extraction of *C.candicans* flavonoids (CCF) based on variables such as solvent, solid-solvent ratio and extraction temperature were evaluated. The optimized conditions were found as Solvent i.e., Aqueous-ethanol (53.42%), Solid-solvent ratio (1:15.83 w/v) and temperature (44.42 °C) and resulted to obtain the TFC as 176.23 mg QRET/g *C. candicans* extract with the yield 27.42 mg CCF/g (*C. candicans* dry weight). LC–MS analysis of CCF, revealed the presence of seven major flavonoids. The antioxidant flavonoids were further used to functionalize the zero-valent silver (ZVAgF) and copper (ZVCuF) nanoparticles. The ZVAgF and ZVCuF were investigated using UV–Vis spectrophotometry, FT-IR spectroscopy and X-ray diffractometry to confirm the presence of the zero valent metals and possible functional groups which capped the elemental metal. Further transmission electron microscopy, dynamic light scattering method and zeta-potential studies were done to understand their respective structural and morphological properties. The efficacy of the as-prepared ZVAgF/ZVCuF as antibiofilm agents on Methicillin-resistant *Staphylococcus aureus* (MRSA) with the mechanism studies have been explored. The MRSA-colony count from the infection zebrafish (in vivo) model, portrayed a reduction of > 1.9 fold for ZVCuF and > twofold for ZVAgF, with no alteration in liver morphology when treated with ZVAgF, implying that the nanoparticles were safe and biocompatible.

## Introduction

*Crotalaria* genus commonly called “rattle pods'' grow in tropical and subtropical regions, and possess one of the largest species of flowering plants^1^. They are widely regarded for their antimicrobial medications in various indigenous traditional systems in Africa, China, and India^2,3^. *Crotalaria* genus are also utilized as a sustainable source in production of biodiesel, biopolymer extraction, feedstock production^4,5^. Phytoconstituents from these plants have been tested for various therapeutic purposes and are considered a sustainable source for flavonoids and other phytoconstituents^6^. *Staphylococcus aureus* is a prevalent foodborne pathogenic microorganism, which invades into the body and causes a wide array of diseases such as skin infections and food poisoning. A more virulent strain of it, Methicillin-resistant *S. aureus* (MRSA) has developed as an extensive root-cause for community- and hospital-acquired infections, worldwide^7^. Upsurge in antibiotic resistance has been compelling, to say the least, and has now become a serious research problem for prospective therapeutic strategies to thwart drug resistance. The application of phyto-molecules as antimicrobials has gained large-scale attention and large numbers of plants have been recorded in traditional systems of medicine and the isolated bioactives from the plants have attracted extensive attention as a new-age, natural antibacterial. Bioactive secondary metabolites from various plant genera, possessing negligible side effects have been in vogue, in traditional medicine, for treating health ailments^8,9^. Myriad phyto-constituents have been well documented as antimicrobial agents, including biofilm inhibition^10,11^. Flavonoids are potent antibacterial agents against a wide range of pathogenic microorganisms and due to antimicrobial resistance (AMR), there is an increasing prevalence of untreatable infections, and flavonoids have gained scientific attention as potential substitutes for antibiotics. Flavonoids and polyphenols have gained a lot of interest from widespread scholars owing to its therapeutic potentials, which include antioxidants and antimicrobials^11,12^.

“Bionanotechnology” has opened new vistas and provides ample research scope in diverse areas, like antimicrobials, anticancer and drug delivery agents^13^. It has been extensively comprehended that the plant extracts reduce metal salts^14^ with the secondary metabolites, which have dual role as stabilizing/reducing agents. The propensity for using plant extracts for functionalizing MNPs is well documented and is reported that MNPs capped with plant extracts offer better antimicrobial efficacy^15^. Silver and copper metals, in zero-valent form are reported to possess, plethora of bioactivity like antimicrobial and anticancer agents, and have encouraged many research groups in bionanotechnology to pursue biogenic synthetic procedures for antimicrobial metallic nanoparticles^16,17^.

*Crotalaria candicans* Wight & Arn, is a native species in Nilgiris, India and is used by the local population as antimicrobials^2^. The studies on phytochemical and bioactivities of these plants hitherto not much explored. In this regard, we assessed the process for valorization of flavonoids of *C. candicans*, establish its antioxidant capacity and nano-conjugate the flavonoids on zero-valent silver and copper (ZVAgF/ZVCuF), with an intent to enhance the antibacterial and antibiofilm activities against MRSA. The functionalized zero-valent nanoparticles were further mechanistically explored to gain insights on the antibacterial and antibiofilm potential, in an in vivo zebrafish infection model.

## Materials and methods

### Plant collection

Plant material used in the study complies with relevant institutional, national, and international guidelines and legislation. The present study complies with the IUCN Policy Statement on Research Involving Species at Risk of Extinction and the Convention on the Trade in Endangered Species of Wild Fauna and Flora, and is not in the red list of IUCN. The *C. candicans* (leaves and stem) were obtained near Kothagiri, Tamil Nadu, India, April 2019. The plant [voucher specimen of herbarium (CARISM 00160)] was certified by Dr. Natesan Ravichandran, SASTRA Deemed University, India. The shade-dried plant parts (750 g) were pulverized and stored in an airtight container.

### Solvent selection

The extraction efficiency with green single solvents—ethanol and isopropanol along with binary solvent mixtures, isopropanol-water and ethanol–water in various proportions were tested for total phenol, total flavonoid, and extraction yield.

### Qualitative analysis for phenolics and flavonoids

The amount of total phenolics was determined using the Folin–Ciocalteu method as reported in the earlier method^18^. Total flavonoid content was estimated based on the aluminum chloride method following the standard procedure^19^.

### RSM optimization for extraction

RSM based Box-Behnken Design (BBD) was used for experimental design for process optimization of enrichment of flavonoid extraction. Ethanol in water (%Solvent) [A], Solid-to-Solvent ratio [B] and Temperature [C] were taken for the optimization. With (A, B, C) as three factorials and three levels (− 1, 0, + 1), in total 17 sets of experiments were established. Triplicate experiments were performed and the obtained mean-values were reasoned out for the total flavonoid content (response). The obtained results were fitted into a second order-polynomial model, with the regression coefficients, Eq. (1).

1 $$Y = \beta_{0} + \sum_{i = 1}^{k} \beta_{i} X_{i} + \sum_{{i = 1}}^{k} \beta_{ii} X_{i}^{2} + \sum_{i = 1}^{k-1}\sum_{i < j = 2}^{k}\beta_{ij} X_{i} X_{j}$$

where the predicted response is Y, intercept as β0, βi, βii, βij (regression coefficients for linear factors) and Xi, Xj (regression coefficients for square of the factors), regression coefficients for interaction effects respectively.

### LC–MS analysis

LC–MS/MS profiling was done with UPLC (Dionex) annexed to a mass spectrometer (microOTOF-Q II, Bruker, Germany). 10 μl of CCF (5 mg/ml) was injected and analyzed in negative (M-H) electrospray ionization modes. A discontinuous gradient elution was done using mobile phase A (CH_3_CN) and mobile phase B (deionized water acidified with CH_3_COOH (1%)). The total run time was 40 min, with increasing % of A and the range of B was (95–0%). The plausible chemical structure of the phytochemicals [CCF] was predicted with the MS/MS pattern available in standard databases and literature^20^.

### Antioxidant activity

#### DPPH free radical scavenging assay and ferric reducing power

Stable free radical-DPPH scavenging ability by the *C. candicans* flavonoids (CCF) and CCF functionalized nanoparticles were determined using the reported method^21^. Different concentrations of ascorbic acid and the extract was prepared in methanol. Having methanol as blank solution, the DPPH free radical scavenging activity was expressed as the percentage of inhibition using the formula

$$\% {\text{scavenging}} = {\text{Control}} - {\text{Sample}}/{\text{Sample}}*{1}00$$

For ferric reducing power assay (FRAP) procedure as described^22^ was used. Antioxidant activity was expressed as absorbance vs Concentration of standard/sample.

### Synthesis of zerovalent silver (ZVAgF) and copper nanoparticles (ZVCuF)

#### CCF functionalized AgNPs optimization

To find the influencing/determining factors for biogenic ZVAgF synthesis, the CCF (2–4 mg/mL); AgNO_3_ molarity (5-15 mM) and Sunlight exposure time (60–120 min) were considered. Out of three factors, two were always constant and the required factor was varied. The characteristic peak of ZVAgF was observed with a spectrophotometer at specific time-intervals of 60, 90 and 120 min. The optimized zerovalent silver (ZVAgF) was further characterized and used for microbiology.

#### CCF functionalized CuNPs optimization

For the CuNPs synthesis, the CCF concentration (2–4 mg/mL); Cu(CH_3_COO)_2_H_2_O[CAM (5–15 mM)] and the reducing agents [RA-NH_4_OH and N_2_H_4_] were used at different volume combinations. The concentration of reducing agents were used as RA1(NH_4_OH-5µL, N_2_H_4_-10µL), RA2(NH_4_OH-10µL, N_2_H_4_-10µL) RA3(NH_4_OH-10µL, N_2_H_4_-5µL) The as prepared, characterized zero-valent copper (ZVCuF) was retained for microbiological studies.

### Characterization of functionalized metallic nanoparticles

UV–VIS spectrophotometer (Lambda 25, PerkinElmer, Waltham, MA) was used to find the formation of ZVAgF/ZVCuF by monitoring in the 350–700 nm wavelength range. FT-IR Spectrometer (Spectrum 100, Perkin-Elmer, New Jersey, USA) was used in the range of 4000–400 cm^−1^, to identify the potential functional groups in the CCF, which were responsible for capping and reducing the elemental Ag/Cu. Powder X-ray diffraction (PXRD) diffractograms were done with Ultima [III] diffractometer (Rigaku, Tokyo, Japan) with Cu-Ka radiation in a 2-h timeframe with 10–80 kV. ZVAgF/ZVCuF were dispersed in polystyrene cuvettes and with dynamic light scattering coupled Zeta Sizer Nano-series (ZS-90 Red, Malvern Instruments, Malvern, England), the particle size/zeta potential analysis was done. The morphology and size of nanoparticles was analyzed using Hi-Resolution Transmission Electron Microscope (HRTEM) (JEOL Japan, JEM-2100 Plus).

### Antimicrobial studies

#### Bacterial strains

For these studies, MRSA—Methicillin resistant *Staphylococcus aureus* [ATCC43300] were maintained at − 80 °C as 15% glycerol stocks, and sub-cultured onto LBA/TSA plates for experiments. The LB broth was used to inoculate the isolated MRSA colonies.

#### Antibacterial screening

The Minimum inhibitory concentration (MIC) and the minimum bactericidal concentration (MBC) were assessed for both bacteriostatic/bactericidal effects of flavonoids capped AgNPs/CuNPs with micro-broth, two-fold dilution method^23^.

#### Time kill kinetic assay

For deducing the bactericidal potential of ZVAgF/ZVCuF, a time kill study as described by Lotha et al.^24^, was performed. The MRSA inoculums were diluted and the cells allowed to grow, in a sterile broth till 10^6^ CFU/mL. Treatments were started with 1X MBC of ZVAgF/ZVCuF and retrieval of cultures at different time points (0–24 h) was done and serially diluted. The colonies were then plated on LB agar plates and determination of plate counts were done for untreated/treated samples.

#### Alizarin red-Q assay

The quantity of Cu^2+^ released from ZVCuF in both absence/presence of MRSA was evaluated with Alizarin Red-Cu^2+^ complex, OD at 510 nm, as described by^25^.

### Studies on biofilm inhibition

#### Crystal-violet (CV) staining and fluorescent imaging

MRSA culture with 0.05 OD was administered on glass slides in a sterile environment in BHI broth with both treatments (with and without 6 μg/mL ZVAgF/2 μg/mL ZVCuF of 1X MIC). After incubation (24 h), the glass slides were washed and dried at ambient temperatures. 0.1% of crystal violet (CV) was used to stain the formed biofilms; excessive CV was washed and air dried^26^.

Similarly for the fluorescent microscopic imaging, the above-mentioned procedure for MRSA biofilm formation was rigorously followed and subsequently was administered with both biogenic ZVAgF/ZVCuF at respective 1X MIC (6 μg/mL for ZVAgF and 2 μg/mL for ZVCuF). 24 h of incubation was done and PBS was used to clear the non-adherent cells and the slides were then tinted with fluorescein diacetate (FDA)/propidium iodide (PI) [1:1 mixture]. Microscopic imaging, CV and FDA/PI was performed using a Nikon microscope (Nikon Eclipse Ni–U, Japan) and Nikon microscope (Nikon Eclipse Ts2)^27^.

#### SEM imaging

For scanning electron microscopy, MRSA cultures diluted to 0.05 optical density were allowed to get attached to a sterile cover glass surface, which was placed inside a microtiter plate (24 wells). Cover glass was inundated with sterile BHI broth and incubated. After incubation with ZVAgF/ZVCuF of 1X MIC (6 μg/mL for ZVAgF and 2 μg/mL for ZVCuF), the unbound cells were irrigated with sterile PBS, fixed with 2% glutaraldehyde, and air-dried. A series of ethanol washes (50–100%) were performed on the biofilms for dehydration. After dehydration, the dried biofilms were sputter-coated with Pt, and the SEM micrograph was obtained using FE-SEM, JEOL 6701F (Tokyo, Japan)^28^.

### Membrane permeability studies

MRSA (mid-log phase) was subjected to centrifugation; the cells were washed twice with PBS, and again re-suspended in PBS of equal volumes. Permeability index was determined with the procedure as reported in literature^29^, with ZVAgF/ZVCuF.

### Bacterial cell surface hydrophobicity (BATH) assay

The BATH assay is usually assessed by finding the partitioning capability of cells, between an aqueous phase and non-aqueous phase (hexadecane). These surface hydrophobicity changes experiments were done by a reported procedure in literature^30^ upon treatment with ZVAgF and ZVCuF.

### Reactive oxygen species (ROS) assay

MRSA when treated with Sub-MIC of ZVAgF and ZVCuF induced the reactive oxygen species (ROS). Dichloro-dihydro-fluorescein diacetate, a fluorophore (at Ex 485 nm and Em 538 nm), gets reduced to dichlorofluorescein by the in-situ generated ROS. Fluorescence changes in the treatment groups and control were quantified using a spectrofluorometer (JASCO FP-8500, JASCO, Tokyo, Japan)^31^.

### Toxicity studies

#### In vitro hemolysis assay

Hemolysis assay was performed by Blood agar diffusion method to measure the toxicity of CCF, ZVCuF and ZVAgF. Briefly, Blood agar plates were prepared by adding 5 mL blood from a healthy adult volunteer to 100 mL of Nutrient agar. 6 mm diameter wells were punched on the agar base and 50 μL of MIC and Sub MIC concentrations of CCF, ZVCuF and ZVAgF were added to the wells. 20% Triton-X 100 was taken as positive control and PBS was taken as negative control. The plates were incubated at 37 °C for 18 h and hemolytic zones were analyzed.

#### In vivo zebrafish model studies

All procedures involving Zebrafish were approved by the Institutional guidelines and carried out following ARRIVE guidelines. The in vivo experiments were approved by the SASTRA Deemed University Institutional Animal Ethics Committee vide CPCSEA-493/SASTRA/IAEC/RPP.

#### Zebrafish toxicity study design

*Danio rerio*, ~ 5 cm in length, aged 2 months, and weighing ~ 300 mg (irrespective of sex), was acquired from a local aquarium in Vallam, Tamil Nadu, India. Using recognized protocols, the acclimatization of fish was done^32^. To deduce the toxicity of the ZVAgF/ZVCuF, 5 fish each were exposed to 1× MBC of the MNPs/doxycycline for 48 h. After exposure, the fish were anesthetized with Tricaine mesylate and euthanized by decapitation. The tissues were collected from all fish (within each group) and homogenized with a Tris–HCl buffer (pH 7.4, 0.1 M). The homogenate was centrifuged and the supernatant was subjected to liver *α* and *β*-carboxylesterase enzyme activity^33^, acetylcholinesterase (AChE) activity^34^ and protein quantification with established protocols^35^.

### Histological studies

All fish (n = 5) from each group were euthanized with ice-cold water, and at the end, were fixed with buffered 10% formalin solution for 24 h, and finally embedded with paraffin wax. 5 μm thickness sections were cut and stained with both hematoxylin and eosin and progressed to imaging for histological studies^36^.

### In-vivo zebrafish infection studies

MRSA culture was diluted [0.05 OD] with media and 10 µl was syringed as an intramuscular injection with a 3/10-cc U-100 insulin syringe with a 0.5-in-long, 29-gauge needle. Post-infection, after 3 h, for 1× MIC studies, 10 µl of zero-valent ZVAgF/ZVCuF were administered near the infection site, and were grouped as treated. Some fish were left untreated and all fish were grouped (n = 5) as either “infected-treated” and “infected untreated”^37^. The anesthetization and euthanization procedures as described above were rigorously followed and the dissected muscle tissues were homogenized with sterile PBS; and plated onto LB agar plates, with serial dilution to quantify the microbial load in respective groups.

## Statistical analysis

All experiments were carried out in triplicates and the results were stated as the mean ± SD. Analysis of variance (ANOVA) followed by Duncan’s Multiple Range Test was made to declare the implication between the tested groups. A value of *p* < 0.05 was considered as statistically significant. The statistical analyses were attained in GraphPad Prism software. RSM-based model-fitting and statistical analysis were obtained by using Design Expert (release 9.0.3.1; State-Ease, Inc., Minneapolis, MN, USA).

## Results

### Effect of solvent on yield, phenolics and flavonoids

Based on the yield analysis, it was clear that the ethanol–water binary mixture was a suitable greener solvent mixture for the extraction of flavonoids from *C. candicans* (Table 1). The total phenolic contents (TPC) (393.82 ± 0.19 mg GAE/g extract) of *C. candicans*, and the total flavonoid contents (TFC) of *C. candicans* (142.52 ± 0.74 QRTE/g extract) with 70% aqueous-ethanol were found significantly higher as compared to other solvent fractions. The total extract yield was 23.24 mg/g with ethanol–water system, for the dry weight of *C. candicans*.

**Table 1:** Selected solvents and its influence on yield, Phenolic, flavonoid content.

Solvent system	Extraction yield (mg/g DW)	Total phenolics (mg GAE/g extract)	Total flavonoids (mg QRTE/g extract)
Ethanol	18.09	308.72 ± 0.95	96.14 ± 0.45
Isopropyl alcohol (IPA)	11.17	213.39 ± 0.77	80.04 ± 0.63
Ethanol–water (7:3)	23.24	393.82 ± 0.19	142.52 ± 0.74
Ethanol–water (1:1)	21.48	382.07 ± 0.94	131.28 ± 0.53
IPA-water (7:3)	15.66	282.66 ± 0.19	84.72 ± 0.67
IPA-water (1:1)	18.91	303.54 ± 0.38	89.39 ± 0.42

*DW* dry weight, *GAE* Gallic acid equivalent, *QRTE* quercetin equivalent, *IPA* isopropyl alcohol.

### RSM optimization

Extraction parameters in a combined interaction have a varied effect than the single factors^38^. Thus, the process of extraction needs to be optimized for effective enrichment of the analytes^39^. In this regard, RSM based BBD has been well utilized and reported to achieve the desired goal^40^. Box-Behnken matrix comprising extraction factors i.e., variable and TFC (QRTE mg/g extract) i.e., response is depicted in Table 2. The polynomial regression model (second order) (Eq. 1) was used to analyze the interaction between the independent variables. An empirical relationship of the dependent variables’ response on TFC (QRTE mg/g extract) with the ethanol-in-water/Solid-to-Solvent ratio/extraction temperature (independent variables), obtained with the coded factors, is depicted in Eq. (2).

2 $$\begin{aligned} {\text{TFC }}\left( {{\text{mg}}\,{\text{QRTE/g}}\,{\text{extract}}} \right) & = 174.09 + 2.19* \, A + 1.60 \, * \, B - 0.73 \, * \, C \\ & \quad - 3.00 \, * \, A \, * \, B + 0.70 \, * \, A \, * \, C - 3.84* \, B \, * \, C \\ & \quad - 7.39* \, A^{2} - 4.75 \, * \, B^{2} - 7.65 \, * \, C^{2} \\ \end{aligned}$$

**Table 2:** Box-Behnken design of the independent variables and experimental results for the response variables.

Experiments	Variables			Response
	Solvent (%)	Solid-solvent (1 g:ml)	Temperature (°C)	TFC (mg QRTE/g extract)
1	30	15	50	156.23
2	30	15	45	165.24
3	70	15	50	162.35
4	50	10	45	166.94
5	50	10	40	158.44
6	30	10	40	144.67
7	30	15	40	157.08
8	50	10	50	162.01
9	50	20	40	169.32
10	30	10	45	155.72
11	70	20	45	162.52
12	50	20	50	158.44
13	50	15	45	173.91
14	70	10	45	165.92
15	70	15	40	159.97
16	30	20	45	163.88
17	50	20	50	158.27

**Figure 1:**
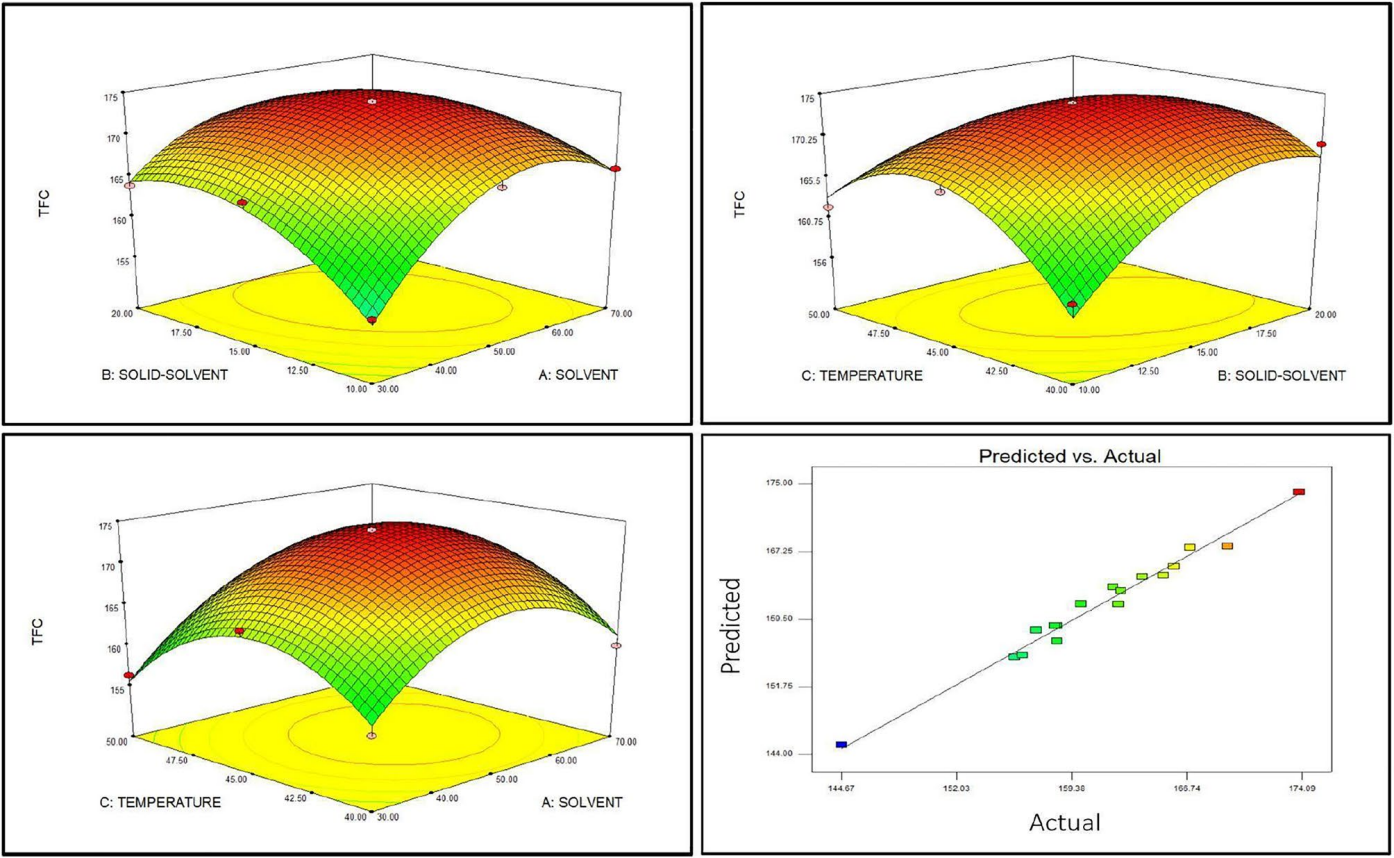
Response surface plots depicting the interaction of extraction parameters (variables) and response.

The significance in the fitness of the model statistics and quadratic nature was assessed by the analysis of variance (ANOVA) results show F-value for model is 39.36, implying that the model is significant with *p* < 0.0001 (Table 3). The model validation was done using the numerical methods, involving correlation coefficient (R^2^) and the adjusted R^2^ (R^2^ adj). The high R^2^ coefficient depicts that the generated quadratic model from the experimental data is acceptable. The results of ANOVA and Regression coefficient are summarized for the TFC obtained (Tables 3, 4). The R^2^ value is 0.98 and adj. R^2^ is 0.95 shows the significance of the model. The coefficient of variation (CV) was 0.85%, confirming that the predicted regression model is precise. The 3D interactive parameter plots (Fig. 1) exhibited the interplay of both variables upon each other, as a response. Optimum conditions for extraction (predicted and obtained) are shown in Table 5 along with its response. The yield of the extract also increased (27.42 mg CCF/g DW).

**Table 3:** ANOVA for Response surface quadratic model.

ANOVA for response surface model				
Source	*df*	Sum of squares	Mean square	F value
Model	9	667.014	74.11	39.36***
A-SOLVENT	1	44.40	44.40	23.58**
B-SOLID-SOLVENT	1	26.20	26.20	13.91**
C-TEMPERATURE	1	4.85	4.85	2.58^#^
AB	1	39.83	39.83	21.15**
AC	1	2.18	2.18	1.16^#^
BC	1	76.27	76.27	40.51**
A^2^	1	166.62	166.62	88.49***
B^2^	1	67.24	67.24	35.71**
C^2^	1	202.99	202.99	107.81***
Residual	7	13.18	1.88	
Lack of fit	6	13.17	2.19	151.85^#^
Pure error	1	0.01	0.01	
Total error	16	680.19		

*p < 0.05, **p < 0.001, ***p < 0.0001 significant; ^#^Not-significant.

**Table 4:** Regression coefficients of the predicted second-order model for the response variables.

Model parameters	Regression coefficient	*df*	S.E
Intercept	174.09	1	1.09
A-SOLVENT	2.19	1	0.45
B-SOLID-SOLVENT	1.60	1	0.4
C-TEMPERATURE	− 0.72	1	0.45
AB	− 3.00	1	0.6
AC	0.70	1	0.65
BC	− 3.83	1	0.60
A^2^	− 7.39	1	0.79
B^2^	− 4.75	1	0.79
C^2^	− 7.65	1	0.74

*S.E* standard error.

**Table 5:** Optimum conditions obtained from response surface modeling and one variable at a time method and its response.

Variable name	Optimum values obtained	
	Response surface modelling	One variable at a time
Solvent (%)	53.42	50
Solid-solvent (1 g:ml)	15.83	15
Temperature (°C)	44.42	45
Observed values of TFC	176.23	173.91
Predicted value of TFC	174.34	–

### Identification and quantification of flavonoids

By LC–MS profiling of the optimized extract CCF- seven major flavonoids, namely; Kaempferol-3-O-sambubioside, Fisetin, Quercetin, Morin, Quercetin 3-O-glucuronide, Kaempferol 3-O-glucuronide and Quercetin 3-O-(caffeoyl)-glucoside were identified and quantified, based on its MS/MS fragmentation profiles and comparing with the literature (Figs. S1–S2).

### Antioxidant activity of the CCF

Free radical scavenging ability of CCF was assessed by DPPH free radical scavenging assay, which revealed that CCF were strong antioxidants in nature as compared to standard ascorbic acid. The %inhibition of radical reduction was observed from 70 to 88% for CCF and to ascorbic acid which is 69–85% respectively for 10–100 μg/mL (Fig. S3A). In a dose dependent manner, ferric reducing power was also observed for CCF (Fig. S3B) for the concentration 10–100 μg/mL.

### Synthesis and optimization for ZVAgF and ZVCuF

**Figure 2:**
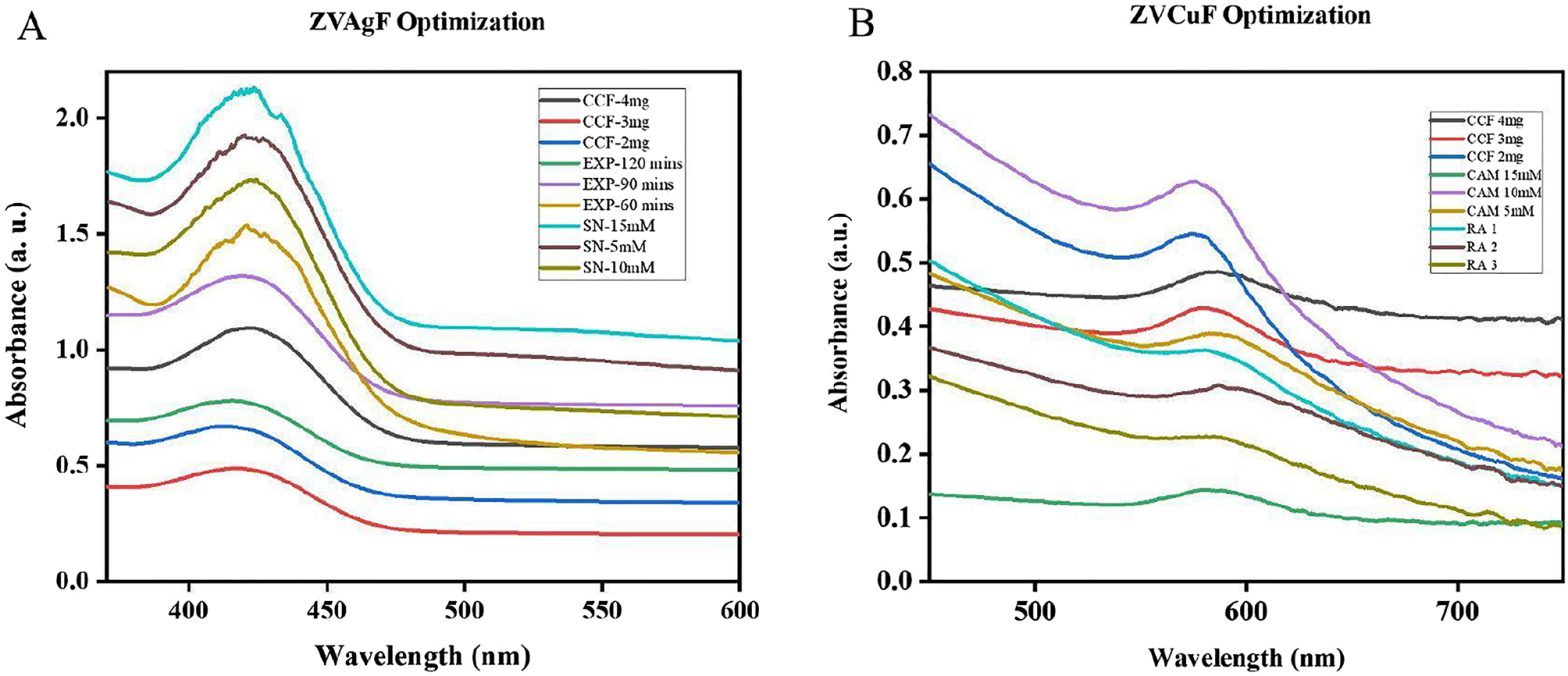
UV–VIS spectrum of optimization studies for the synthesis of CCF capped AgNPs/CuNPs. (**A**) ZVAgF, (**B**) ZVCuF.

The zero-valent silver biogenic synthesis was done by determining its wavelength (350-600 nm) and optical-density (OD) with UV–visible spectroscopy. For the ZVAgF synthesis, the CCF concentration was aliquoted to 2–4 mg/mL; AgNO_3_ concentration was in the range of 5, 10, 15 mM. The time exposure to sunlight was in the range of 60 min to 2 h. CCF 2 mg/ml concentrations added to AgNO_3_ solution 10 mM AgNO_3_ produced stable zero-valent silver under sunlight-exposure time of 90 min (Table 6), characterized with the optical-density (λ_max_) at 423 nm (Fig. 2A). For the synthesis of CuNPs functionalized with CCF (ZVCuF), the CCF concentration aliquoted to 2–4 mg/mL; and CAM concentration was in the range of 5, 10, 15 mM and Reducing Agents, NH_4_OH 5-10µL, N_2_H_4_ 5-10µL. Out of these different parameters CAM 10 mM, NH_4_OH-10µL, N_2_H_4_-10µL produced stable ZVCuF. On further optimization with CCF concentration 2 mg/mL of CCF exhibited zero-valent copper nanoparticles (Table 6) and characterized with the optical density (λ_max_) at 580 nm (Fig. 2B).

**Table 6:** Optimized variables for synthesis of silver/copper nanoparticles and their particle size.

ZVAgF		ZVCuF	
CCF concentration (mg/mL)	2	CCF concentration (mg/mL)	2
AgNO3 concentration (mM)	10	Copper acetate monohydrate (mM)	10
Sunlight exposure time (min)	90	NH4OH (µL), H6N2O (µL)	10
particle size	77	Particle size	106

### Characterization of ZVAgF/ZVCuF

**Figure 3:**
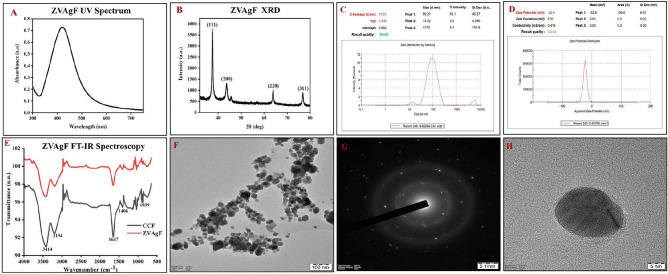
Physicochemical characterization of ZVAgF (**A**) UV-VIS Spectrum (**B**) XRD (**C**) Zeta-sizer (**D**) Zeta-potential (**E**) FT-IR Spectrum, TEM Analysis of ZVAgF (**F**) 100 nm (**G**) SAED (**H**) 5 nm.

ZVAgF absorbance λ_max_ was found at 423 nm with UV–Vis spectroscopy (Fig. 3A). From the PXRD diffractogram, in accordance with the JCPDS, File No. 4-0783, the 2θ values of elemental silver was found at 37.53°, 47.78°, 63.87°, 76.76° are attributable to the (111), (200), (220) and (311) FCC planes (Fig. 3B). The size average of the ZVAgF was ~ 77 nm, as evidenced from the zetasizer data (Fig. 3C). The zeta potential value of − 22.8 mV, indicated that the zerovalent silver nanoparticles are very stable (Fig. 3D). TEM imaging exhibited the morphology (Fig. 3F) spherical shape of ZVAg (Fig. 3H). Selected Area Electron Diffraction (SAED) revealed the crystalline structure of ZAgF (Fig. 3G).

**Figure 4:**
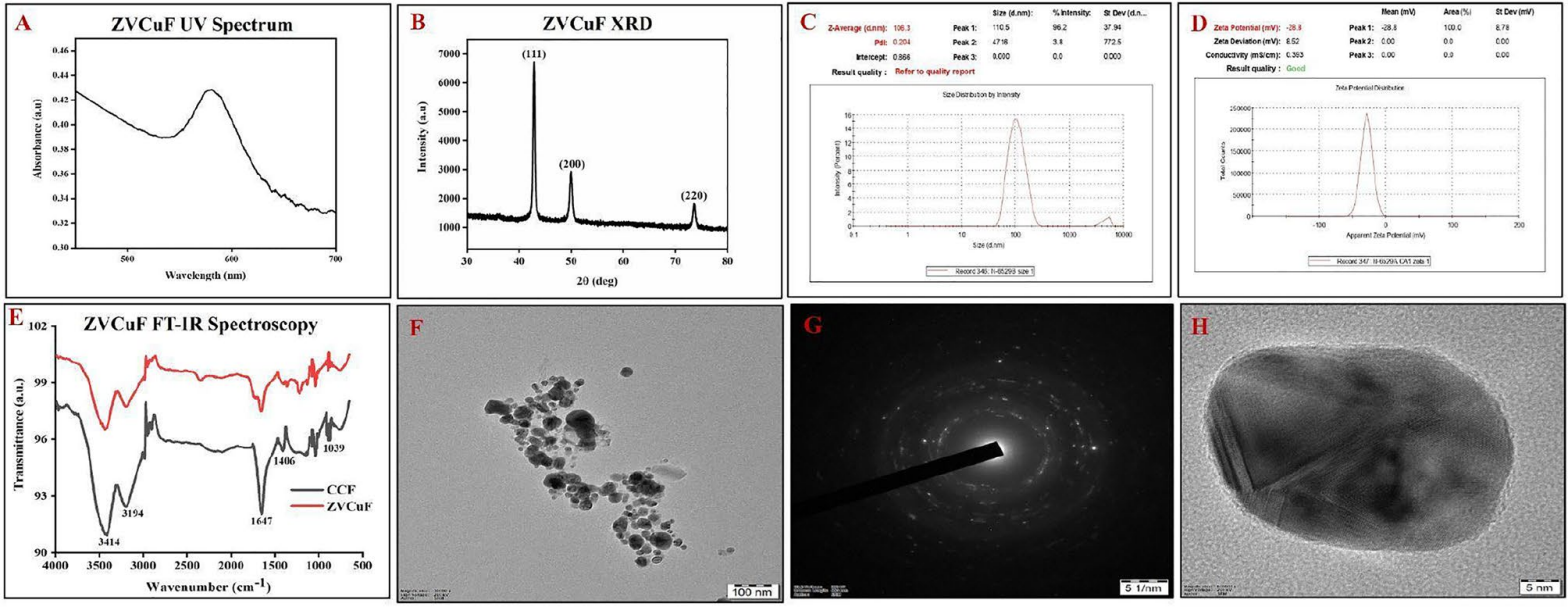
Physicochemical characterization of ZVCuF (**A**) UV-VIS Spectrum (**B**) XRD (**C**) Zeta-sizer (**D**) Zeta-potential (**E**) FT-IR spectrum TEM Analysis of ZVCuF (**F**) 100 nm (**G**) SAED (**H**) 5 nm.

The surface plasmon bands were deciphered at λ_max_ 580 nm for ZVCuF, confirming the presence of elemental copper (Fig. 4A). The crystalline nature of the zerovalent copper was unequivocally proved with the recorded diffraction peaks at 42.86°, 49.94°, and 73.6° attributable to the face-centered cubic indices (111), (200), and (220) respectively (Fig. 4B). zero-valent copper had an average size of ~ 106 nm (Fig. 4C). The zeta potential measurement of -28.8 mV confirmed the high stability of ZVCuF (Fig. 4D). TEM imaging exhibited the morphology (Fig. 4F) spherical shape of ZVCuF (Fig. 4H). Selected area electron diffraction (SAED) revealed the crystalline structure of ZCuF (Fig. 4G) Fourier Transform Infrared Spectroscopy (FT-IR) exhibited the bond linkages and functional groups of CCF, which were capped on ZVAgF and ZVCuF. The major IR absorption bands were at 3500–3100 and 1600–1400 cm^−1^ and could be attributed to the stretching vibrations of O–H, C═C, C–H and C–O groups, respectively of the flavonoids in CCF (Figs. 3E, 4E).

### Antioxidant activity of ZVAgF/ZVCuF

Free radical scavenging ability of ZVAgF and ZVCuF was compared with CCF. The % inhibition of ZVAgF and ZVCuF was not effective as CCF. Radical reduction was observed from 58 to 68% at 10–100 μg/mL range of ZVAgF and ZVCuF exhibited 34–66% at 10–100 μg/mL range (Fig. S3).

### Effect of ZVAgF and ZVCuF on antibacterial/antibiofilm activity

The MIC/MBC values of *C. candicans* extract, CCF, ZVAgF, ZVCuF and standard (Doxycycline) are presented in Table 7. CCF was found to have an MIC value of 64 µg/mL against MRSA and the MBC/MBIC was at 256/128 µg/mL respectively. However, with the zerovalent silver and copper functionalized with CCF (ZVAgF/ ZVCuF), an enhanced antibacterial activity was observed. ZVCuF displayed its respective MICs/MBCs at 2/32 µg/mL, while for ZVAgF it was at 6/64 µg/mL. The crystal violet assay portrayed that the ZVAgF and ZVCuF had the capacity to inhibit the MRSA biofilms and the Minimum Biofilm Inhibitory concentration (MBIC) was found to have 8 and 4 µg/mL respectively and it can be thus inferred that both the zerovalent silver and copper had antibiofilm effects sub-MIC values. Prima facie, it can be thus inferred that the ZVAgF and ZVCuF had the propensity to exhibit true antibiofilm effect, meriting further exploratory studies on MRSA.

**Table 7:** Screening of flavonoids enriched fraction of *C. candicans* and capped metallic nanoparticles against MRSA.

Secondary metabolites (SM)	Secondary metabolites/extract	CCF capped AgNPs [ZVAgF]	CCF capped CuNPs [ZVCuF]
	MICs/MBCs/MBICs (µg/mL)		
*Crotalaria candicans extract*	> 400	–	–
CCF	64/256/128	6/64/8	2/32/4
Doxycycline	1/16/2	–	–

### Time kill kinetics

The bactericidal effect of zero-valent Ag/Cu on MRSA, was determined by colony counts at various intervals (0–24 h). Both the MNPs effected a substantial reduction of > 1.6/ > 2 log CFU, whereas standard drug Doxycycline treatment caused significant 2.7 log reduction in CFU (Fig. S5A).

### ARS-conjugation assay

ARS-conjugation assay, showed that the presence of bacterial cells (MRSA) led to an enhanced release of Cu^2+^ from ZVCuF, relative to Cu (II) release observed in a medium, devoid of cells. The above observation (Fig. S5B), implies that the interaction of NPs with the cell-surface of MRSA, augments the copper ions release from ZVCuF in 24 h which could be attributed for the enhanced antibacterial/antibiofilm potential of ZVCuF against MRSA.

### Antibiofilm effect mechanistic studies

**Figure 5:**
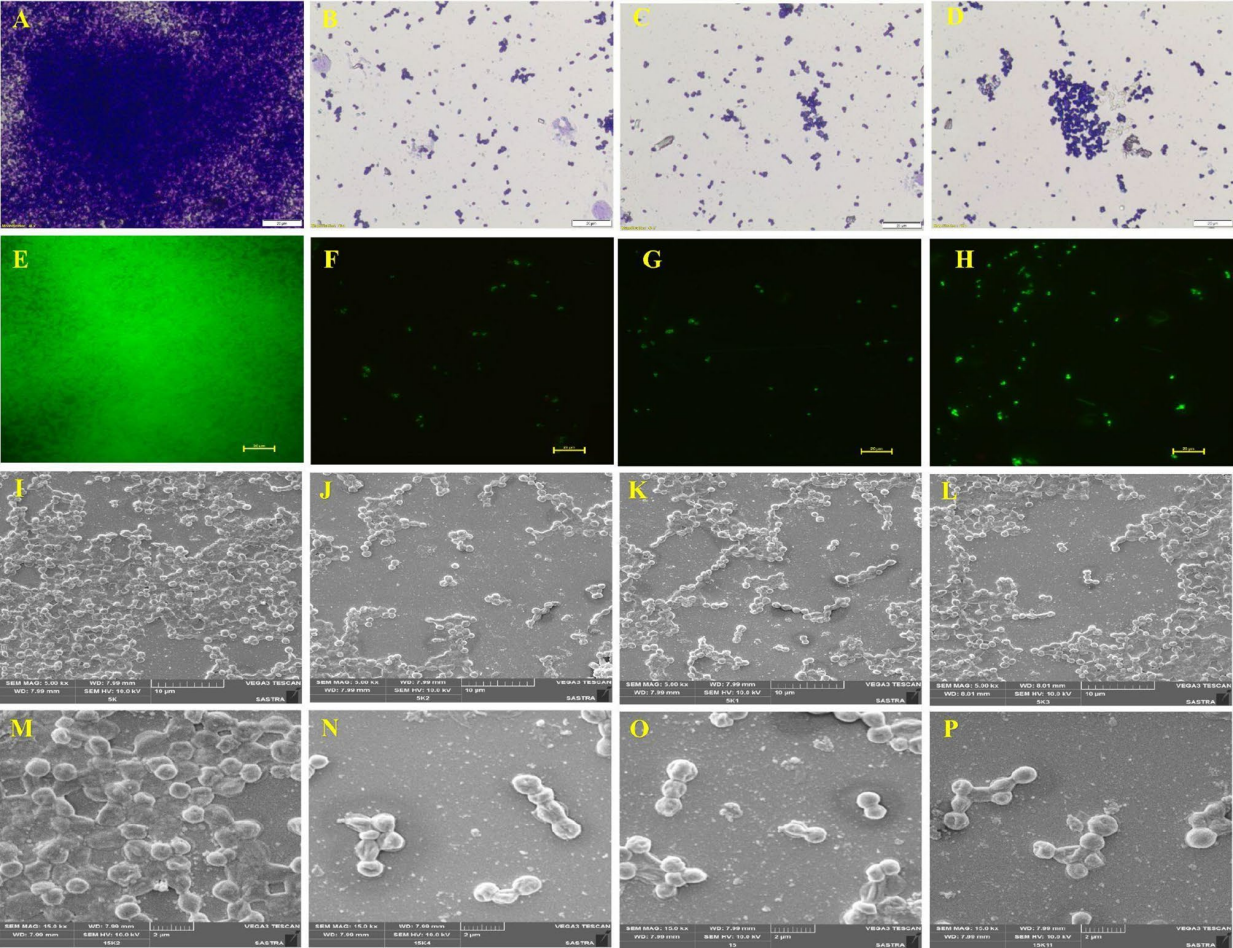
Crystal violet, fluorescent imaging and SEM imaging of MRSA biofilm with CCF and CCF capped nanoparticle. Crystal Violet (**A**) Untreated-MRSA biofilms (**B**) 1× MIC ZVAgF treated (**C**) 1× MIC ZVCuF treated and (**D**) 1× MIC Doxycycline treated. Fluorescence Live-Dead Imaging of biofilms formed on glass surface (**E**) Untreated, (**F**) 1× MIC ZVAgF treated, (**G**) 1× MIC ZVCuF treated, and (**H**) 1× MIC Doxycycline treated images.SEM imaging of MRSA biofilm treated with ZVAgF and ZVCuF. Unhindered colonization of untreated bacteria (**I**, **M**). 1× MIC ZVAgF treated (**J**, **N**), 1× MIC ZVCuF treated (**K**, **O**), and 1× MIC Doxycycline (**L**, **P**) treated images. Magnification @10 µm, 2 µm respectively.

Imaging of biofilms by Light microscopy (Crystal Violet) (Fig. 5A–D), Fluorescent microscopy (Fig. 5E–H) and SEM (Fig. 5I–P) showed 1× MIC concentration of zero-valent Ag/Cu, had the potential to restrict the biofilm formation. Based on these encouraging results, the possible mechanism(s) behind the antibiofilm potential of flavonoids capped zero-valent Ag/Cu, in presence of propidium iodide and CTAB (positive control) were explored. Propidium Iodide is extruded by metabolically active cells and is accumulated in metabolically inactive cells; accumulation of propidium iodide depicts an increased membrane permeability, when mediated by ZVAgF and ZVCuF^41^. The fluorescence ratio between untreated, and CTAB treated cells, known as Permeability index (%PI) was expressed. Treatment of MRSA with ZVAgF and ZVCuF caused a 30–45% rise in membrane PI, relative to untreated control (Fig. S6A), which might contribute to the antibiofilm potential of nanoconjugates^42^. Reactive oxygen species (ROS) induces damage on cells, which might also lead to impaired biofilm formation. The quantification of ROS was done with DCHFDA assay and the results revealed that ZVAgF and doxycycline treatment led to increased ROS release when compared to ROS generated by untreated control groups whereas ZVCuF did not trigger ROS production (Fig. S6B). As ROS can lead to loss in cell viability and as, a notable proportion of dead cells was not observed with fluorescent staining, ROS contribution to the antibiofilm effects of ZVAgF and ZVCuF is limited. ZVAgF had a 35% decline in Cell surface hydrophobicity (CSH), whereas a 50% decline triggered in CSH was found with ZVCuF. Doxycycline treated group did not induce any change, implying that treatment with both ZVAgF and ZVCuF reduced adherence and might hinder the biofilm formation by MRSA^43^ (Fig. S6C).

### Toxicity studies

#### In vitro hemolysis assay

The Hemolysis assay was performed to evaluate the toxicity of CCF, ZVCuF and ZVAgF (Fig. S7). CCF, ZVAgF and ZVCuF did not display hemolytic potential at MIC and Sub MIC concentrations implying that the NPs were non-toxic (non-hemolytic).

#### Toxicity studies in zebrafish model

Liver carboxyl esterase and brain acetylcholinesterase (AchE) are notable markers to determine the toxicity of zero-valent Ag/Cu on zebrafish. AchE is important for metabolism of acetylcholine and other choline-esters and is a biomarker in neuronal toxicity studies^44^. Liver carboxylesterase (CEs) plays an important detoxification part in the use of xenobiotics. Elevated carboxylesterase levels, due to xenobiotic stress, trigger the breakdown of carboxylic-esters and are deemed as significant components of liver detoxification system^45^. The ZVAgF, ZVCuF and the positive control drug effects on liver carboxylesterase and brain acetylcholinesterase profiles were determined. Significant differences were not observed between the treated and control groups, when analyzed for liver carboxylesterase with α-naphthol (Fig. S8A) and β-naphthol (Fig. S8B) assays. Brain acetylcholinesterase levels revealed that there was no significant difference among the treatment and control groups. (Fig.S8C). Therefore, treatment with biogenic ZVAgF/ZVCuF did not significantly change the brain and liver enzyme profiles, relative to the control which underscores their non-toxic nature.

### Studies on histopathological changes in zebrafish liver

**Figure 6:**
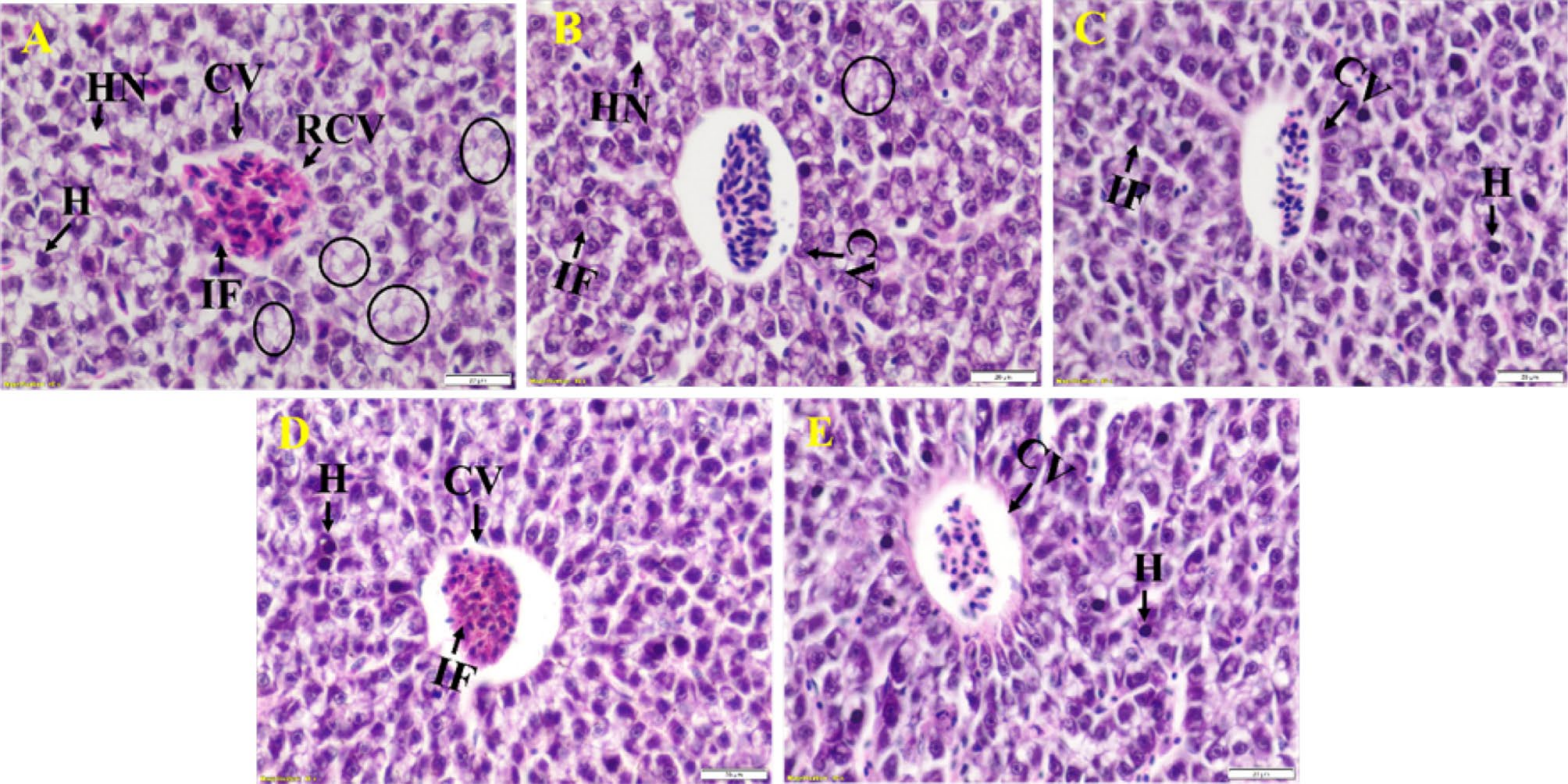
Histopathology changes in the liver of Zebrafish. Representative image of liver stained with Hematoxylin (purple color-nuclei) and eosin (pink color-cytoplasm). (**A**) MRSA Infected liver showing Central Vein (CV) Cytoplasmic Vacuolation (Round), Hepatocytes (H), Infiltration of Inflammatory cells (IF), Rupture of Central Vein Wall (RCV), areas of hepatic necrosis (HN) (**B**) Doxycycline treated liver exhibiting Infiltration of cell (IF), hepatic necrosis(HN) (**C**) ZVAgF treated liver showing Central vein (CV), Hepatocytes (H), Infiltration of Inflammatory cells (IF) and (**D**) ZVCuF treated liver showing Central vein (CV), infiltration of cells inside the central vein, Hepatocytes (H) (**E**) Untreated liver showing normal structure of hepatic tissue and arrangement of hepatocytes Central Vein (CV), Hepatocytes (H).

Histopathological analysis was done to elucidate the effect of ZVAgF and ZVCuF. MRSA infected zebrafish liver shows high infiltrated lymphocytes, hepatic necrosis and cytoplasmic vacuolization (Fig. 6A). Treatment with Doxycycline leads to infiltration in inflammatory cells, and cellular swelling. In addition, hepatic necrosis was also observed (Fig. 6B). Upon treatment with ZVAgF despite maintaining overall liver architecture, lymphocyte infiltration in the sinusoid, along with inflammatory cell accumulation in the central vein, was observed (Fig. 6C). Treatment with ZVCuF showed mild scattered inflammation and infiltration of inflammatory cells in the central vein, (Fig. 6D), relative to untreated control portraying, no alteration in liver morphology (Fig. 6E). Therefore, despite mild inflammation that was observed in histopathological analysis, unaltered liver and enzyme profile shows that biogenic MNPs are safe (Fig. S9).

### In vivo infection study

**Figure 7:**
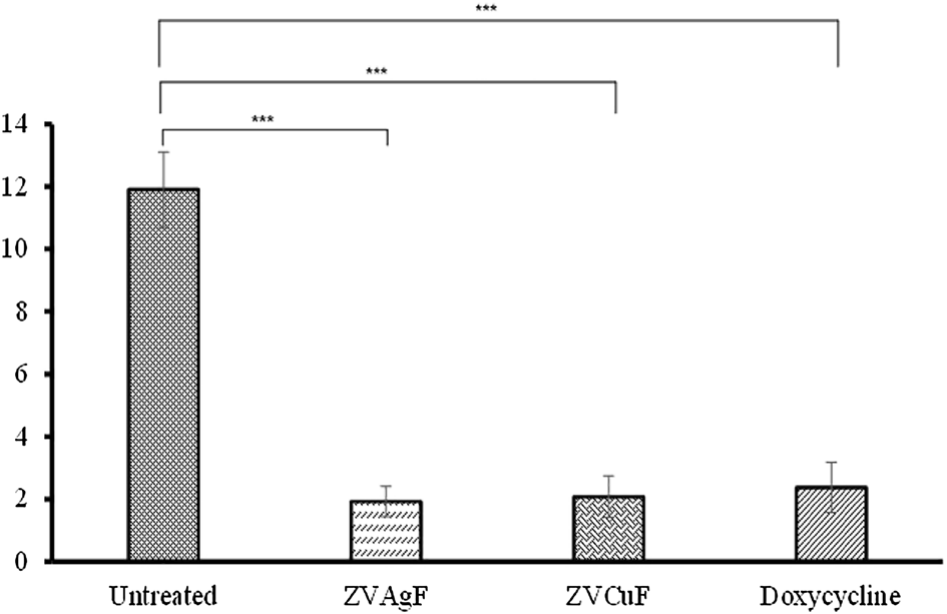
Infection study on Zebrafish model. Colony counts after treatment with ZVAgF, ZVCuF and Doxycycline. ***p < 0.0001.

Zebrafish (*Danio rerio*) possess > 80% genetic similarity with humans and hence are deemed as a model for studying human infectious agents^46^. In vitro antimicrobial/ anti-biofilm potential of biogenic AgNPs and CuNPs against MRSA, encouraged us to further explore its activity in an *in-vivo* infection study. Infecting the Zebrafish with MRSA followed by treatment with ZVAgF and ZVCuF and Doxycycline showed a significant cell count decline by ~ 1.9 log (Fig. 7), in relation with untreated control. Interestingly, decline in the bacterial bioburden was observed at the sub-MIC concentration, with zero-valent Ag/Cu. It is plausible that treatment with biogenic metal NPs stimulates innate immunity of zebrafish and aids in enhanced pathogen clearance by macrophages and neutrophils, which could be attributed for the reduction in bioburden was observed, with sub-MIC concentrations.

## Discussion

The available reports in the literature suggest that the antimicrobial properties of the *Crotalaria* genus are majorly due to its flavonoid content^47,48^. In this regard, we have utilized *C. candicans* to isolate flavonoids by a RSM optimization. From the preliminary analysis of solvents in extraction efficiency, it was clear that ethanol–water was a suitable greener solvent system for extraction of flavonoids from *C.candicans* based on the yield analysis, total phenolic [TPC] and flavonoids [TFC] analysis (Table 1) which is in concurrence with other reports^49^. Using the BBD approach, the yield of the CCF was increased to 27.42 mg CCF/g DW along with TFC as 176.23 mg QRTE/g extract, when compared with the preliminary binary solvent extraction procedure (142.52 mg QRTE/g extract). The model was well validated using statistical analysis and testing the optimized conditions of extraction. The presence of seven flavonoids in the valorized mixture was confirmed with LC–MS analysis (Fig. S1) and it was observed that the content of flavonoids such as Quercetin and Morin together constituted nearly 78% (based on peak area) and other flavonoids were less than 10% each (Table S1). Antimicrobial potency of secondary metabolites has been proven by various research groups^50–52^ and flavonoids are one among them, possessing interesting chemical structural motifs. These flavonoids are reported to possess various biological functions like anticancer, neuroprotective antidiabetic and majorly as antioxidants^53–56^.

In the green synthesis of metal ions to zero valent state using plant extract or extract fractions, it is well reported^57^ that reducing nature of metal ions is due to redox reactions^58^. While a plethora of reports are available with plant extract mediated biogenic synthesis of MNPs, still very few reports are available with enriched/quantified extracts and individual secondary metabolites. The benefit of using quantified plant metabolites like CCF for fabricating nanoparticles [NPs], embraces the ability to predict mechanism of formation of NPs and hypothesize the role of plant metabolite in biological activity. The CCF at 2 mg/ml, reduced silver and copper to zero valent silver and copper nanoparticles respectively. The antioxidant property of the CCF had a great effect on functionalizing/reducing both the ZVAgF (Ag^+^) and ZVCuF (Cu^2+^) from their respective salts. The as-prepared ZVAgF/ZVCuF did not portray appreciable antioxidant activity, which corroborated the fact that the functional groups present in CCF were consumed for capping the metallic nanoparticles. As the ZVCuF/ZVAgF portrayed antimicrobial and antibiofilm effects, further optimization for the synthesis of biogenic metallic nanoparticles was done. Influencing factors like valorized CCF concentration, metal salts and sunlight (photo-mediation, as in case of ZVAgF) and reducing agents (with ZVCuF) were deciphered and with the variation of CCF concentration, it was found that the intensity and sharpness (λ_max_) changes was gratifying (Fig. 2). ZVCuF formation was done by varying CCF concentration and with minimal reducing agents (Fig. 2B). UV–Vis spectra absorption maxima for ZVAgF were found at 400–430 nm (Fig. 3A) and for ZVCuF, it was around 570–600 nm (Fig. 4A). The crystalline nature of ZVAgF/ZVCuF was proved with X-ray diffraction (XRD) studies (Fig. 3B/4B). The large negative zeta potential magnitude − 22.8 mV and − 28.8 mV of ZVAgF (Fig. 3D) and ZVCuF (Fig. 4D) respectively indicates the potential stability of metallic nanoparticles and thus proving that the ZVCuF and ZVAgF are colloids that do not have the tendency to aggregate^59,60^. The presence of the functional groups of flavonoids like the O–H, C=C and C–O on the surface of biogenic nanoparticles, ZVAgF and ZVCuF was confirmed by FT-IR spectroscopy and were attributed to the wavenumbers at 3414, 3194, 1647, 1406, 1039 cm^−1^ respectively (Figs. 3E, 4E respectively). The TEM images exhibited the spherical morphology, nature and size of ZVAgF (Fig. 3F–H) and ZVCuF (Fig. 4F–H). We had previously reported that the phytochemical stabilized nanoparticles exerts appreciable antimicrobial and antidiabetic activity^12,61^. Herein, we synthesized the coinage metal stable nanoparticles which were functionalized with flavonoids (ZVCuF/ZVAgF), and were extensively employed for antibiofilm application. While the valorized CCF did not exhibit notable antimicrobial and antibiofilm effects, the zerovalent metallic nanoparticles (ZVCuF/ZVAgF), functionalized with CCF, were active, as evident from the MIC/MBCs/MBICs data (Table 7). The presence of reducing and capping/stabilizing agents, restrict the MNPs from aggregation, resulting in the production of nanoparticles with smaller hydrodynamic radii. The carboxyl groups and the –OH groups of flavonoids are majorly involved in metal capping and the keto-enol tautomerism of the carboxyl (C=O) group helps in reducing the metal ions to metallic NPs^62–65^. The decrease in –O–H concentration in the IR spectrum can be attributed to its reaction with metal salts Ag^+^/Cu^2+^ ions and subsequent conversion to the zero-valent silver and copper (Fig. S4).

The antimicrobial studies of ZVAgF/ZVCuF against MRSA showed that ZVAgF is more effective than ZVCuF in restricting the growth of MRSA with the MIC/MBIC of 6 ug/mL and 8 ug/mL respectively. Time kill curve results (Fig. S5A) implied that bactericidal effects of both AgNPs and CuNPs were comparable to doxycycline for 24 h. The alizarin test confirmed that nano-copper released cations (Cu^2+^) to the growth medium (Fig. S5B) and these Cu^2+^ ions through electrostatic attraction got bound to the bacterial cell walls; or at times penetration of CuNPs into the bacteria, which results in cell death^66,67^. CV and fluorescent imaging proved the ability of biogenic ZVAgF/ZVCuF to thwart the biofilm formation by biogenic ZVAgF/ZVCuF at MIC levels (Fig. 5A–H). The biofilm SEM micrographs (Fig. 5I–P), concluded that the flavonoid capped ZVAgF/ZVCuF retarded the biofilm formation^68^. Membrane perturbation (Fig. S6A) and ROS studies (Fig. S6B) proved that the ZVAgF aids in ROS generation at higher concentration (1× MIC). Earlier studies have shown that Ag^+^ ions are released into the media, to generate ROS and thus triggering the cell death and retarding the biofilm formation^69^. This is in line with other reports that by reducing hydrophobicity, biogenic NPs retards bacterial adhesion thereby curtailing biofilm formation^70^ as observed in the present study (Fig. S6C). The ZVCuF/ZVAgF displayed better in vitro biofilm activity.

The in vitro hemolysis assay and zebrafish model was employed to ascertain the toxicity of biogenic NPs. CCF, ZVAgF and ZVCuF did not display any hemolytic potential at MIC and Sub MIC concentrations (Fig. S7). α and β-naphthol and acetylcholinesterase levels were observed with treatment relative to the untreated control, and no significant variations were found, implying that the coinage metallic nanoparticles are non-toxic (Fig. S8) which shows similar to other reports^71^. An interesting observation from histopathology analysis was that infiltration cells in the central vein was higher for ZVCuF, when compared with the other treated groups, and as the NPs did not induce mortality in fish, nanoparticles can be construed to be non-toxic (Fig. S9). For the fish infection studies, the ZVCuF/ZVAgF were employed at their MIC, and in vivo infection studies revealed that the nanoparticles triggered a substantial reduction in colony count. ZVCuF induced > 1.9 log reduction whereas the ZVAgF caused > 2 log reduction and Doxycycline exhibited > 2.3 log reduction, evidencing that the ZVCuF/ZVAgF displayed similar ability like doxycycline in restricting bacterial growth (Fig. 7).

## Conclusion

In this study, we have attempted to valorize the *C. candicans* for antioxidant flavonoids. A binary solvent mixture of ethanol/water was attempted for the extraction and parameters like total phenolic (TPC) and total flavonoid (TFC) content and were found to be greater with the ethanol/water binary mixture. A constructive Box–Behnken design was developed and the optimum conditions were found to be (53.42%v/v) of aqueous-ethanol, 44.42 °C as temperature and 1:15.83 w/v as solvent to solid ratio, to yield 27.42 mg of CCF/g of *C. candicans* dry weight and TFC as 176.23 mg QRET/g *C. candicans* extract as a response. Further the antioxidants were used to synthesize silver (ZVAgF) and copper (ZVCuF) nanoparticles. The fabricated NPs exhibited bacteriostatic and bactericidal effects on MRSA. The zebrafish (in vivo) toxicity studies, portrayed that there were no significant alterations in brain/liver enzyme levels. Histopathological results concluded that no severe alterations had occurred when treated with ZVAgF. Hence the antioxidant flavonoids of *C. candicans* have great efficacy for functionalization on zerovalent silver nanoparticles and it is also a moral lead and a capable alternative for combating drug-resistant pathogens.

### Supplementary Information

Supplementary Information.
