# Supplementary material for: BBD optimized antioxidants of Crotalaria candicans and its nanoconjugates, exert potent in vivo anti-biofilm effects against MRSA
Source: Sci Rep. 2023 Sep 29;13:16407. doi: 10.1038/s41598-023-43574-0 (PMC10541877; doi:10.1038/s41598-023-43574-0)
Supplement: Supplementary file 1 — Supplementary Information. [file 41598_2023_43574_MOESM1_ESM.pdf]

Supplementary data

BBD optimized antioxidants of *Crotalaria candicans* and its nanoconjugates, exert potent *in vivo* anti-biofilm effects against MRSA

Ramya M. Subramani[a†], Robert Lotha[a†], Bhanuvalli R. Shamprasad[a], Sriram Sridharan[b], Ravichandran Natesan[b], Saisubramanian Nagarajan[c*], Arvind Sivasubramanian[a*]

[a]Department of Chemistry, School of Chemical and Biotechnology, SASTRA Deemed to be University, Thanjavur, Tamil Nadu, India

[b] Centre for Advanced Research in Indian System of Medicine, School of Chemical and Biotechnology, SASTRA Deemed to be University, Thanjavur, Tamil Nadu, India

[c] Centre for Research on infectious Diseases, School of Chemical and Biotechnology, SASTRA Deemed to be University, Thanjavur, Tamil Nadu, India

***Corresponding authors**

[a] Arvind Sivasubramanian, Department of Chemistry, School of Chemical and Biotechnology, SASTRA Deemed To Be University, Thanjavur, Tamil Nadu, India; arvi@biotech.sastra.edu

[c]Saisubramanian Nagarajan, Centre for Research on infectious Diseases, School of Chemical and Biotechnology, SASTRA Deemed To Be University, Thanjavur, Tamil Nadu, India; sai@scbt.sastra.edu

†Both authors contributed equally

Table S1. Characterization of Flavonoids in CCF using LC-MS/MS-QTOF

S.NO	Metabolites	Percentage present (peak area)	Rt (min)	$[M-H]^+$ (m/z)	MS/MS (Product Ion)	Ref.
1	Kaempferol-3-O- sambubioside	2.03	3.14	579.1	285.1, 318.1, 449.1	**Ref- 1-2**
2	Fisetin	6.04	3.37	287.1	121.1, 137.1, 185.1, 285.1	**Mass bank database (BS003351) Ref- 3- 4**
3	Quercetin	54.53	3.45	301.1	105.1, 151.1, 254.8	**Mass bank database (PB004103)**
4	Morin	24.37	3.84	301.0	151.0, 175.1, 227.0, 271.0	**Mass bank database(BS003339) Ref- 5-6**
5	Quercetin-3-O-glucuronide	3.05	3.94	477.1	151.1, 179.1, 227.0, 285.0, 301.1	**Mass bank database(PR100978) Ref-7**
6	Kaempferol 3-glucuronide	2.07	5.7	461.1	152.9, 179.5, 227.3, 285.2	**Mass bank database (PR100967) Ref-7-8**
7	Quercetin-3-O-(caffeoyl)-glucoside	1.92	5.94	625.1	285.1, 301.1, 463.1	**Ref-9-10**

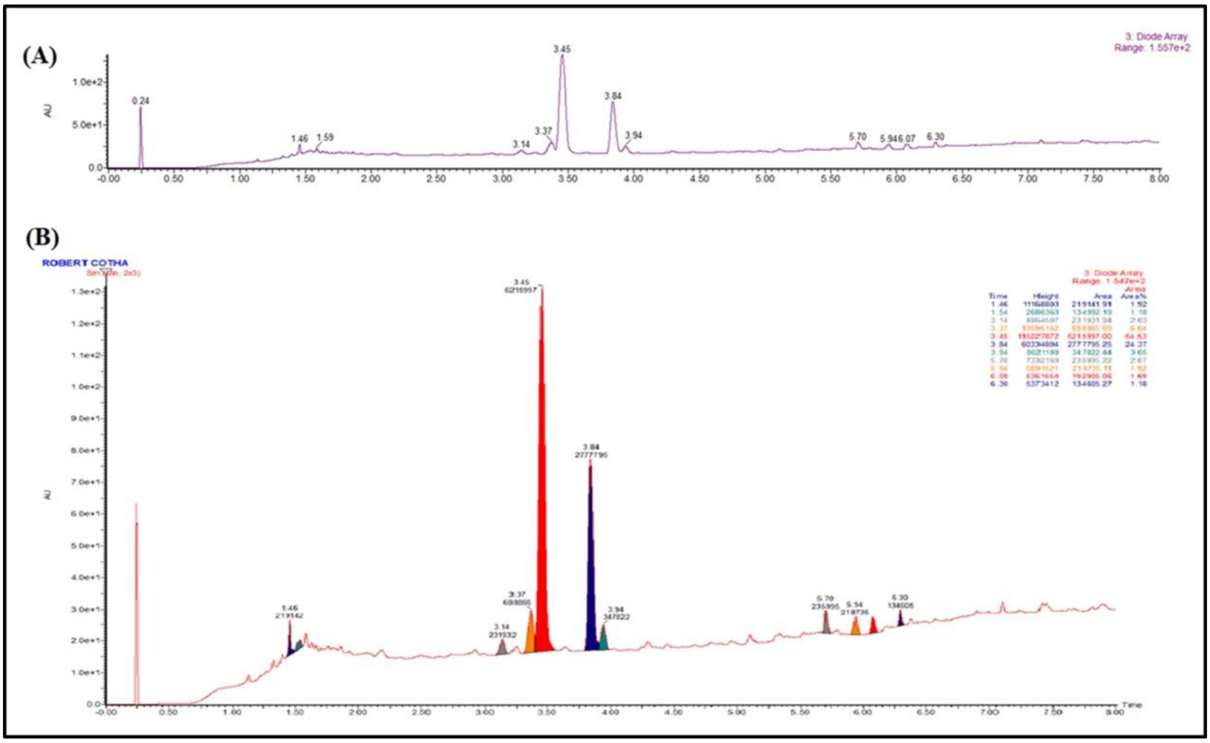

Figure S1: (A) LCMS spectra of the flavonoids enriched *Crotalaria candicans* fraction. (B) Quantitative representation of flavonoids in CCF.

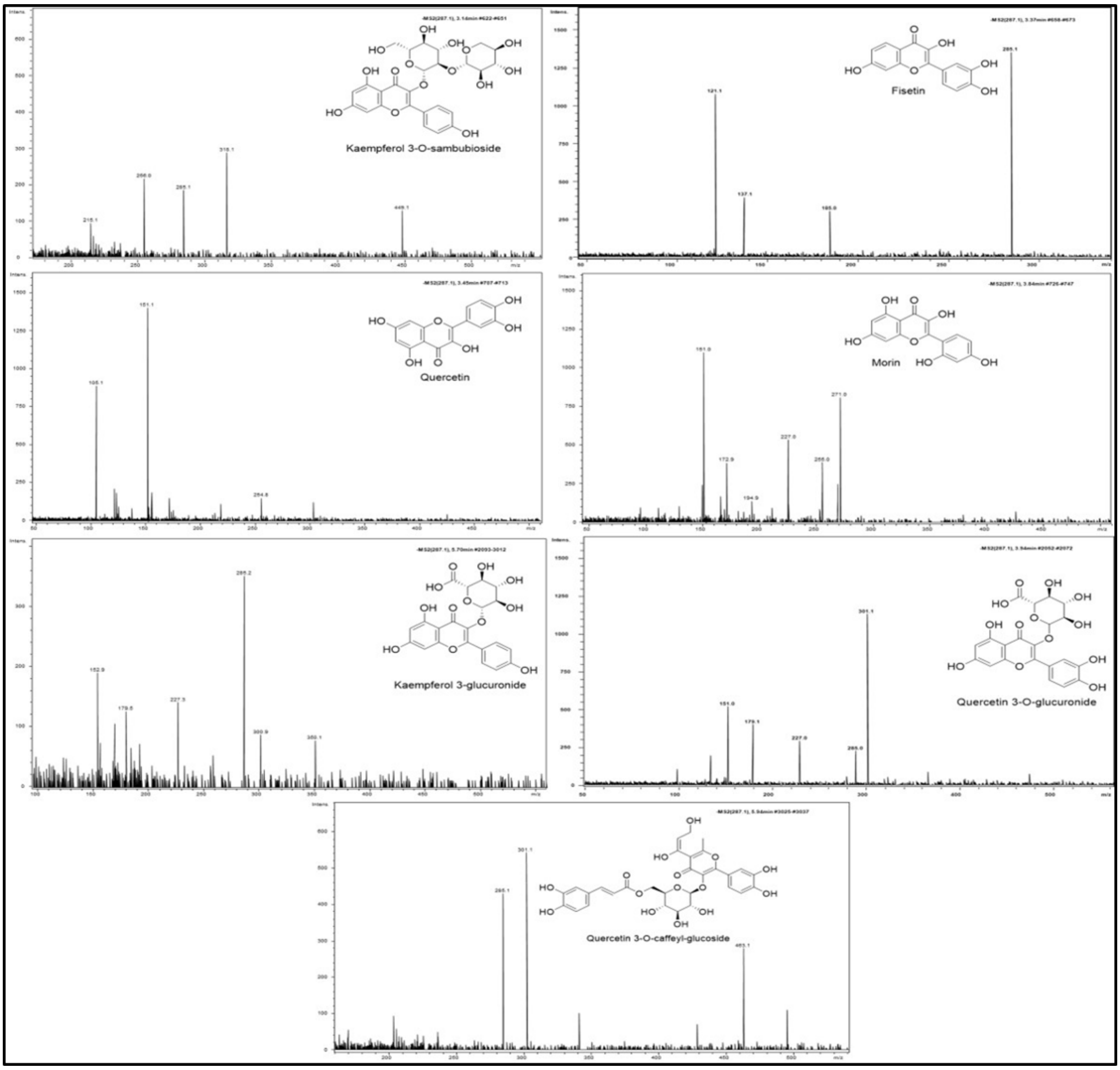

Figure S2: MS/MS profile of the flavonoids enriched from *Crotalaria candicans*.

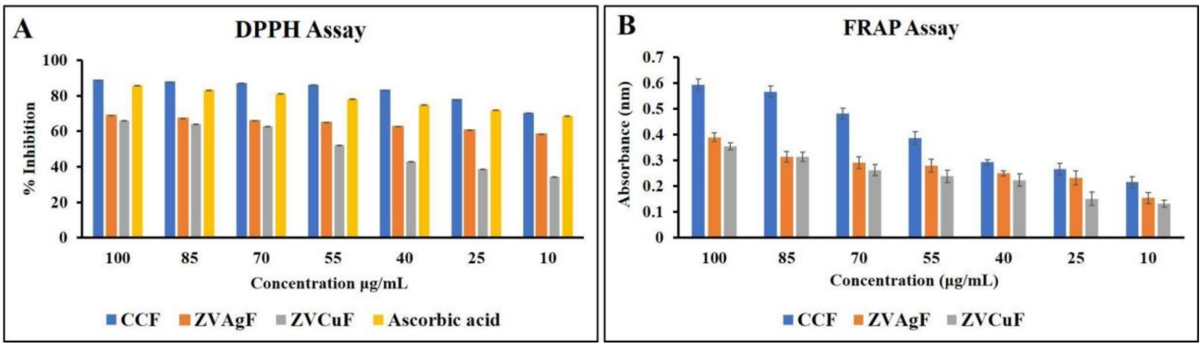

Figure S3: CCF, ZVAgF and ZVCuF Antioxidant Assay : (A) DPPH (2,2-diphenyl-1-picrylhydrazyl) radical scavenging activity (B) Ferric Reducing power

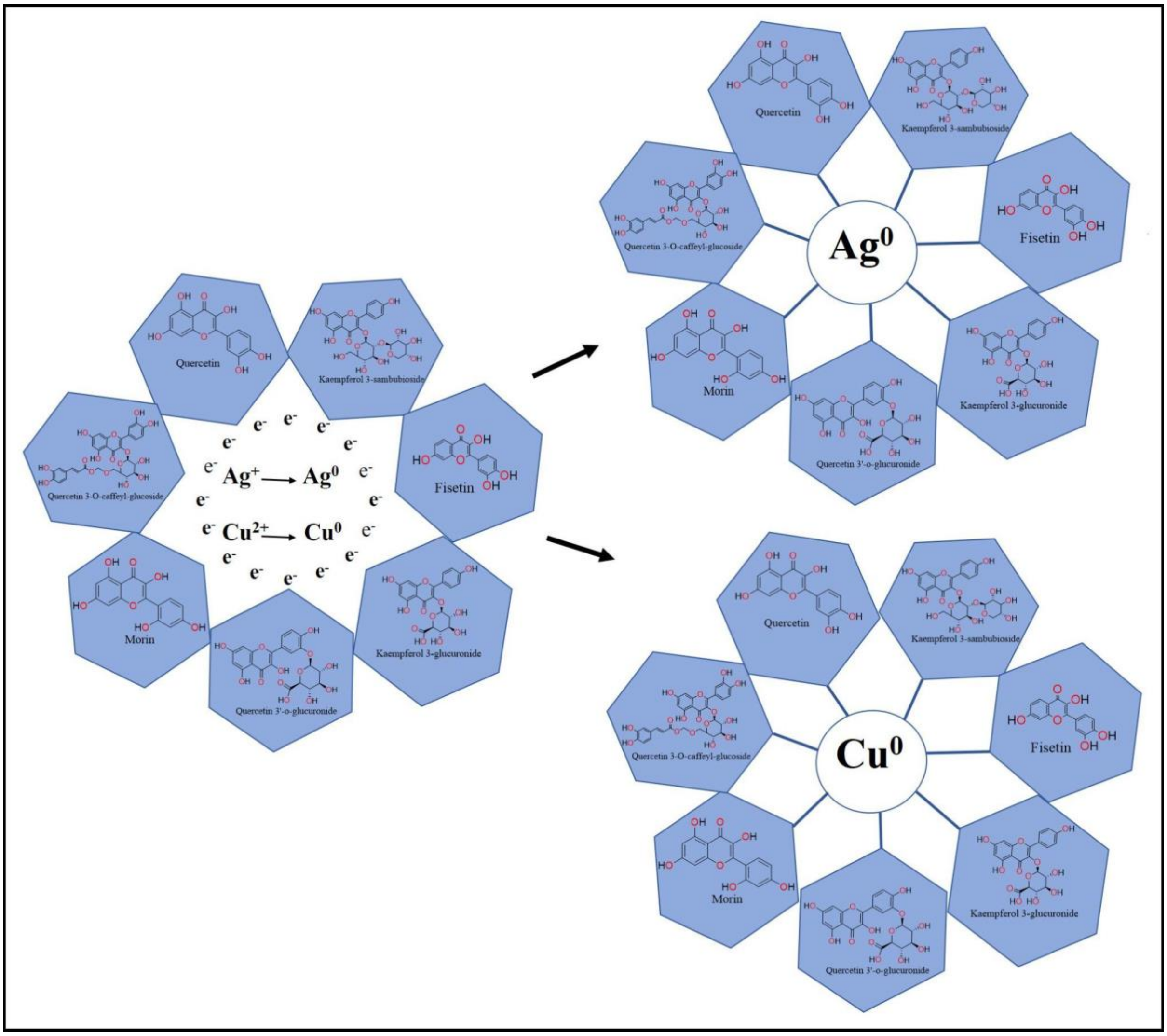

Figure S4: Mechanism of CCF mediated NPs Synthesis

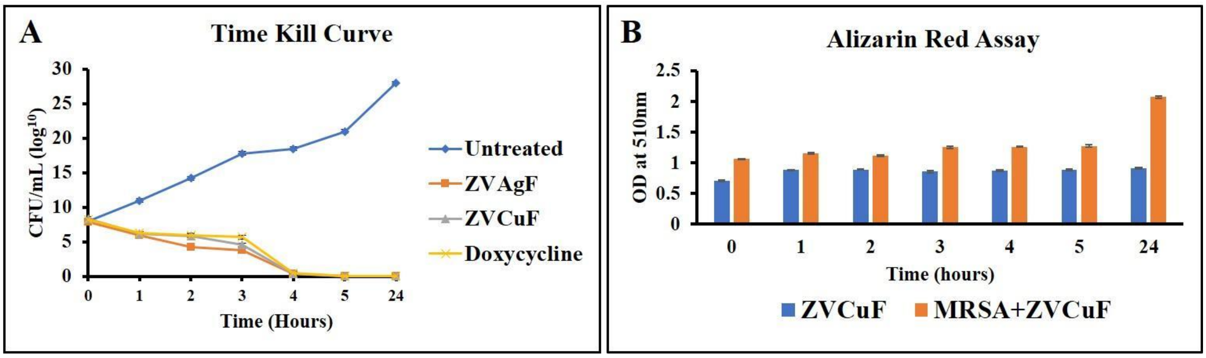

Figure S5. (A) Time kill curve and (B) Alizarin Red Stain (ARS) conjugation test

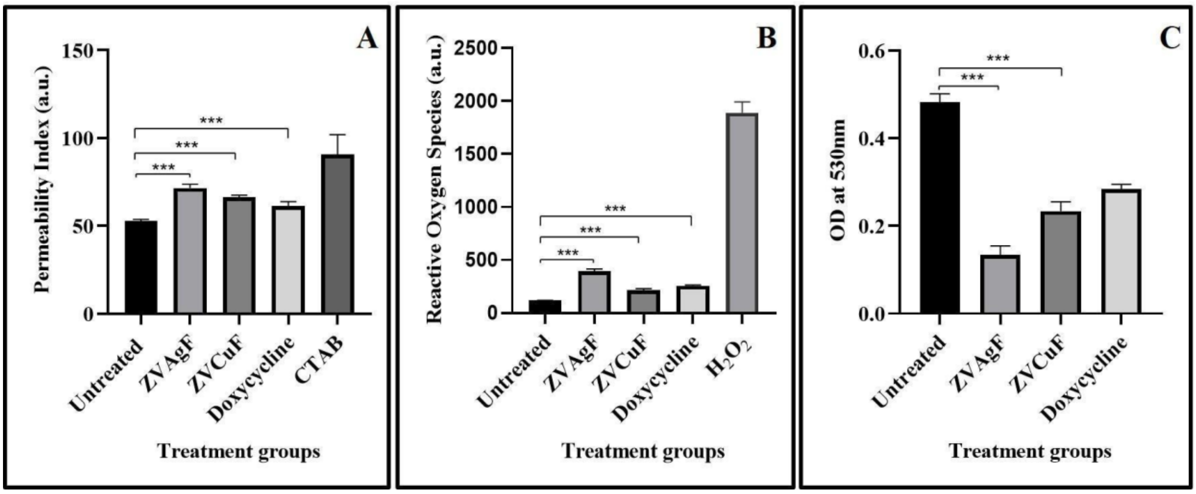

Figure S6: (A) Membrane perturbation, (B)Reactive Oxygen Species and (C)Hydrophobicity Assay

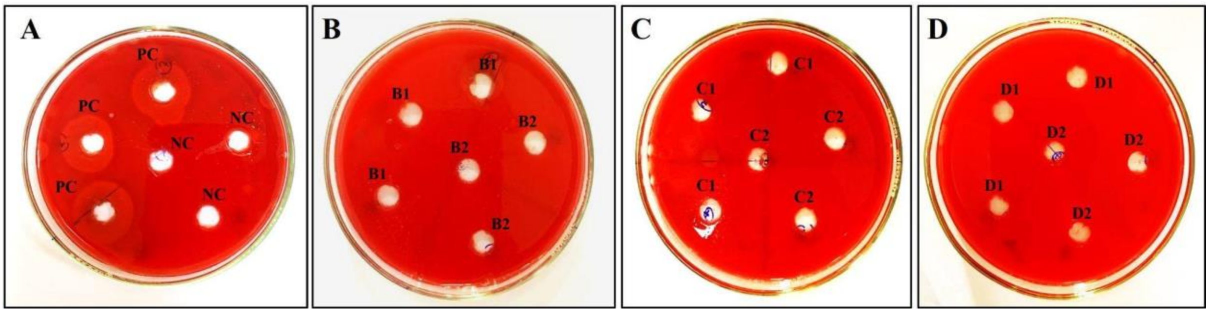

Figure S7. Hemolysis assay with CCF and CCF capped NPs. (A) Triton-X100 Positive control (PC) and PBS Negative Control (NC), (B) CCF (B1 – 64µg/mL, B2- 32µg/mL), (C) ZVCuF (C1 – 6µg/mL, C2- 3µg/mL), and ZVAgF (D1 – 2µg/mL, D2- 1µg/mL)

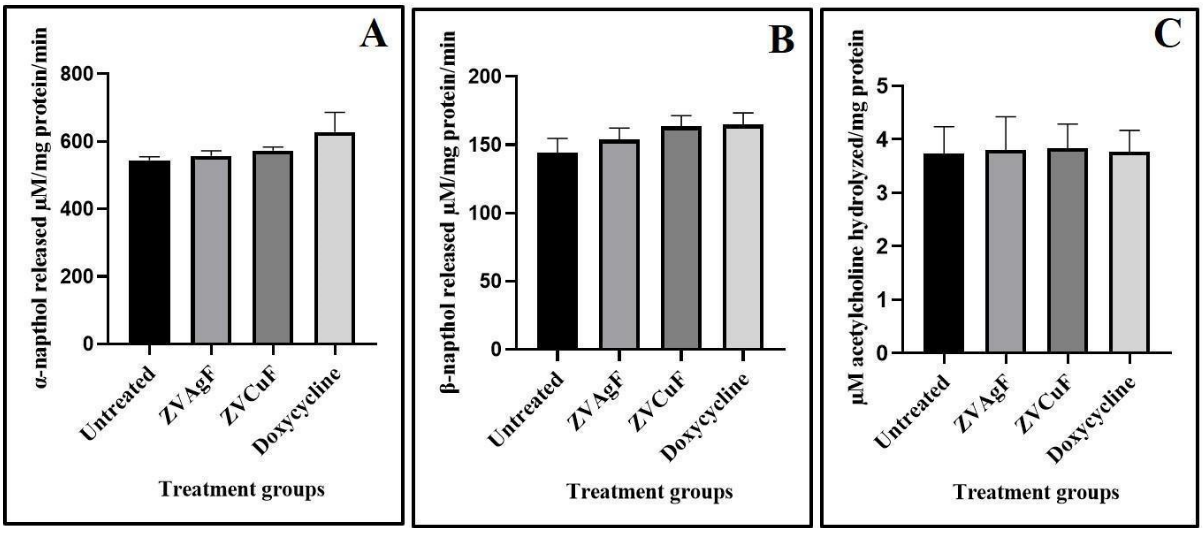

Figure S8: Biogenic CuNPs effect on liver carboxylesterase and brain acetylcholinesterase activity ZVAgF/ZVCuF and Doxycycline on liver carboxylesterase activity A)α-naphthol, B) β-naphthol and on the brain, C) acetylcholinesterase level.

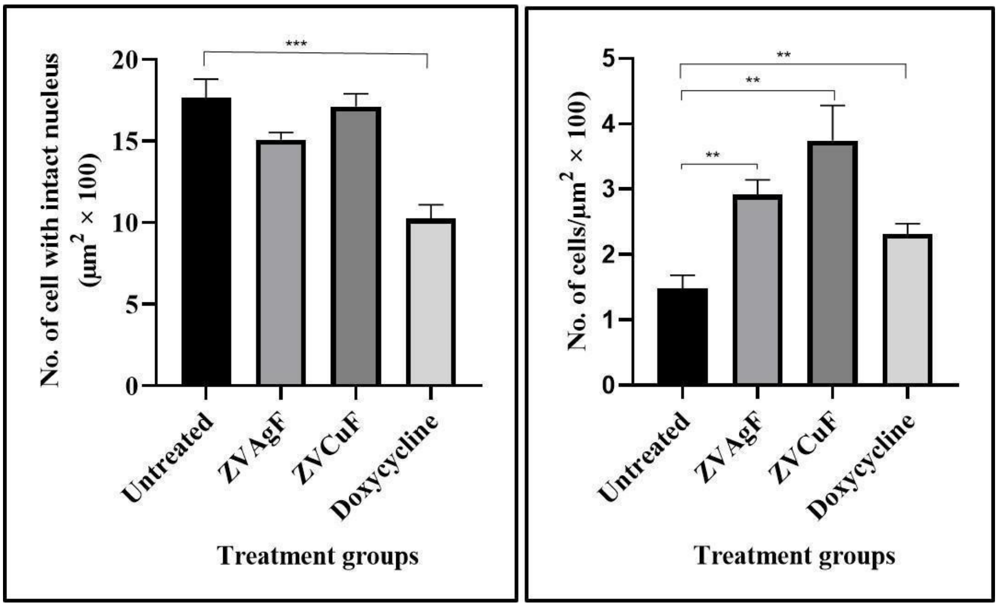

Figure S9: Quantification of normal/ infiltrated cells in zebrafish liver.

References:

1. Qiao, J. *et al.* Phenolamide and flavonoid glycoside profiles of 20 types of monofloral bee pollen. *Food Chemistry* **405**, 134800 (2023). https://doi.org/10.1016/j.foodchem.2022.134800
2. Shi, X., Luo, S., Zhong, K., Hu, X. & Zhang, Z. Chemical profiling, quantitation, and bioactivities of Du-Zhong tea. *Food Chemistry* **394**, 133552 (2022). https://doi.org/10.1016/j.foodchem.2022.133552
3. Jin, M. J. *et al.* A Liquid Chromatography–Tandem Mass Spectrometry Method for Simultaneous Quantitation of 10 Bioactive Components in *Rhus verniciflua* Extracts. *J Chromatogr Sci* bmv152 (2015). https://doi.org/10.1093/chromsci/bmv152

4. Xu, L. *et al.* Enzyme-assisted ultrasonic-microwave synergistic extraction and UPLC-QTOF-MS analysis of flavonoids from Chinese water chestnut peels. *Industrial Crops and Products* **117**, 179–186 (2018). https://doi.org/10.1016/j.indcrop.2018.03.012

5. Wu, D. *et al.* In Situ Study of Metabolic Response of *Arabidopsis thaliana* Leaves to Salt Stress by Neutral Desorption-Extractive Electrospray Ionization Mass Spectrometry. *J. Agric. Food Chem.* **67**, 12945–12952 (2019). https://doi.org/10.1021/acs.jafc.9b05339

6. Pérez-Ramírez, I. F., Reynoso-Camacho, R., Saura-Calixto, F. & Pérez-Jiménez, J. Comprehensive Characterization of Extractable and Nonextractable Phenolic Compounds by High-Performance Liquid Chromatography–Electrospray Ionization–Quadrupole Time-of-Flight of a Grape/Pomegranate Pomace Dietary Supplement. *J. Agric. Food Chem.* **66**, 661–673 (2018). https://doi.org/10.1021/acs.jafc.7b05901

7. Aaby, K., Mazur, S., Nes, A. & Skrede, G. Phenolic compounds in strawberry (Fragaria x ananassa Duch.) fruits: Composition in 27 cultivars and changes during ripening. *Food Chemistry* **132**, 86–97 (2012). https://doi.org/10.1016/j.foodchem.2011.10.037

8. Kumar, S., Singh, A. & Kumar, B. Identification and characterization of phenolics and terpenoids from ethanolic extracts of Phyllanthus species by HPLC-ESI-QTOF-MS/MS. *Journal of Pharmaceutical Analysis* **7**, 214–222 (2017). https://doi.org/10.1016/j.jpha.2017.01.005

9. Vallejo, F., Tomás-Barberán, F. A. & Ferreres, F. Characterisation of flavonols in broccoli (*Brassica oleracea* L. var. italica) by liquid chromatography–UV diode-array detection–electrospray ionisation mass spectrometry. *Journal of Chromatography A* **1054**, 181–193 (2004). https://doi.org/10.1016/j.chroma.2004.05.045

10. Francescato, L. N., Debenedetti, S. L., Schwanz, T. G., Bassani, V. L. & Henriques, A. T. Identification of phenolic compounds in *Equisetum giganteum* by LC–ESI-MS/MS

and a new approach to total flavonoid quantification. *Talanta* **105**, 192–203 (2013).

https://doi.org/10.1016/j.talanta.2012.11.072
